# Sleep disturbance is associated with reduced DTI-ALPS index and altered functional-network efficiency in internet gaming disorder

**DOI:** 10.3389/fpsyt.2026.1892235

**Published:** 2026-10-15

**Authors:** Longyao Ma, Bohui Mei, Mengzhe Zhang, Wenjing Li, Xinyu Wang, Jiawen Tian, Hui Zhang, Yan Lang, Qiyan Lv, Yarui Wei, Shaoqiang Han, Yong Zhang

**Affiliations:** 1 Department of Magnetic Resonance Imaging, the First Affiliated Hospital of Zhengzhou University, Zhengzhou, China; 2 Zhengzhou Key Laboratory of Brain Function and Cognitive Magnetic Resonance Imaging, Zhengzhou, China; 3 Department of Psychiatry, the First Affiliated Hospital of Zhengzhou University, Zhengzhou, China

**Keywords:** diffusion tensor imaging analysis along the perivascular space, glymphatic system, graph theory analysis, internet gaming disorder, mediation analysis, sleep

## Abstract

**Background:**

Sleep disturbance is common in internet gaming disorder (IGD), but its possible involvement in the pathophysiology of IGD remains unclear. Therefore, we aimed to investigate the glymphatic system-related function in IGD patients and to establish the associations among glymphatic system, human brain network, and clinical features.

**Methods:**

This study recruited 66 IGD patients and 35 healthy controls (HC), who underwent magnetic resonance imaging (MRI) scanning and clinical assessment. The glymphatic system-related diffusivity and network efficiency were calculated based on diffusion images along the perivascular space (DTI-ALPS) method and resting-state functional MRI, respectively. Differences between groups, neural/clinical correlations, and mediation analyses were further explored.

**Results:**

Compared with HC, IGD patients showed a significantly lower ALPS index and global network efficiency. The partial correlation analysis showed that the ALPS index in IGD patients was associated with PSQI, HAMA, and HAMD scores. Moreover, the ALPS index partially mediated the association between global brain efficiency and sleep quality in exploratory cross-sectional mediation analysis.

**Conclusions:**

The current study revealed abnormalities of ALPS index and network efficiency in IGD patients and suggested that the cross-sectional associations among global brain efficiency, sleep quality, and ALPS index were consistent with an indirect pathway, which may reveal the neuropathological mechanisms of IGD.

## Introduction

Sleep disturbance, a common comorbidity of internet gaming disorder (IGD), has been reported in more than 60% of IGD patients with long-term circadian rhythm disruption and chronic sleep deprivation (1, 2). Several studies showed that chronic sleep disturbance may increase the risk of neurocognitive disorders, anxiety, and depression (3, 4). Therefore, it is important to explore the neural mechanisms underlying the comorbidity between IGD and sleep disturbance.

The glymphatic system is a brain-wide fluid transport system and facilitates the rapid exchange of cerebrospinal fluid (CSF) and interstitial fluid (ISF) mediated by aquaporin 4 (AQP-4) (5), which is critical for maintaining brain health (6). Notably, the sleep-wake cycle is closely associated with glymphatic fluid transport: sleep increases glymphatic clearance compared to wakefulness (7). Recently, the diffusion tensor image analysis along the perivascular space (DTI-ALPS) method proposed by Taoka et al. has emerged as a putative glymphatic-related diffusion marker (8). The DTI-ALPS reflected the extent of glymphatic system-related dysfunction by quantifying the diffusivity of water molecules along the perivascular space of projection and association fibers at the level of the lateral ventricle (8). In addition, the higher reproducibility of the DTI-ALPS method has been reported (9) and strongly correlated with the regional signal unit ratio measured with the intrathecal injected contrast agent (10). Therefore, the DTI-ALPS method may emerge as a valuable neuroimaging biomarker for investigating the relationship between sleep disturbance in IGD and glymphatic system-related function.

In addition, the effect of sleep disturbance on brain function in IGD patients has become an active area of research. Zheng et al. revealed that sleep disturbance exacerbated the severity of IGD, which was mediated by the hippocampus-caudal nucleus resting state functional connectivity (rs-FC) (11). Zhou et al. and Niu et al. found that rMFG-bilateral PoCG rs-FC and dReHo of the left caudate nucleus were significantly correlated with the total PSQI score, respectively (12, 13). However, the above methods depended on the selection of *a priori* seed regions or synchronization between voxels, which could not evaluate FC among all voxels across the whole brain. In recent years, the graph theory analysis provided new perspectives for multi-scale understanding of the internal correlations of brain networks by quantifying the complex human brain networks into the topology of nodes and edges (14). More than that, poor sleep quality has been shown to negatively impact human brain networks (15).

However, although glymphatic system-related function should be altered in IGD with elevated sleep-disturbance symptoms (IGD-high PSQI) patients theoretically, it has not been quantified. Moreover, the association between functional network efficiency and ALPS index in IGD remains unclear. Therefore, understanding the associations among sleep quality, ALPS index, and human brain networks will be essential to provide insight into the neuropathologic mechanisms of IGD patients.

This study is as follows: First, the DTI-ALPS method and functional MRI (fMRI) were used to assess whether glymphatic system-related diffusivity and human brain networks were impaired in IGD patients; Second, the association between the ALPS index and the human brain network was explored; Third, we examined whether ALPS index can mediate the relationship between human brain networks and sleep quality; Fourth, whether the ALPS index could distinguish between IGD-high PSQI patients and IGD without elevated sleep-disturbance symptoms (IGD-low PSQI) patients. We hypothesized that decreased ALPS index and brain network efficiency were observed in IGD patients, which closely correlated with the severity of clinical features.

## Materials and methods

### Participants

The study was approved by the Ethics Committee of the First Affiliated Hospital of Zhengzhou University (2022-KY-0438). All subjects or guardians signed an informed consent form after being fully educated about the purpose and procedure of the study and were assured of the security and privacy concerning the children’s medical records.

This study recruited 66 IGD patients from the psychiatric outpatient service of the First Affiliated Hospital of Zhengzhou University. Based on the fifth edition of the Diagnostic and Statistical Manual of Mental Disorders (DSM-V) criteria, the diagnosis of IGD was confirmed by an experienced psychiatrist (Yan. L). The severity of all IGD patients was assessed by another experienced psychiatrist (Qiyan. L) using the Young’s Internet Addiction Test (IAT). Sleep quality over a month period was assessed in all IGD patients using the Pittsburgh Sleep Quality Index (PSQI), which is a self-report questionnaire and consists of 19 items and 7 components. The total score ranges from 0 to 21, with lower scores indicating better sleep quality. Therefore, prior to the inclusion of subjects, we also carefully questioned IGD patients about the presence of sleep disturbance and the onset time to determine that the sleep disturbance developed after IGD and lasted for at least one month. In addition, all IGD patients must also fulfill at least 5 of the DSM-V and the IAT score ≧ 50. To comprehensively assess the clinical characteristics of IGD patients, we also collected gaming time (hours/week), illness duration, HAMD scores, and HAMA scores. This study also recruited 35 age-, gender-, and education years-matched healthy controls (HC) from the community. All subjects were Han Chinese and right-handed. Exclusion criteria for all subjects were as follows: (1) the person or their first-degree relative with a formal clinical diagnosis of severe neurodegenerative/neuropsychiatric disorders (Alzheimer’s disease, Parkinson’s disease, Anxiety disorder, and Major depression disorder); (2) recent history of sedative, analgesic, or narcotic medications; (3) serious organic brain lesions or somatic diseases (brain tumors, traumatic brain injury, diabetes, and kidney disease); (4) subjects with a maximum head motion > 2.5 mm or rotation > 2.5°. The participant flow diagram in **Supplementary Figure S1**.

### MRI protocols

All subjects’ MRI data were obtained using a 3.0-T GE MRI scanner equipped with an 8-channel receiver array head/neck coil (Discovery, MR750w, Milwaukee, WI, USA). All subjects were instructed to close their eyes, not shake, stay awake, and not think about anything before the scan. All subjects were equipped with earplugs/headphones and sponge cushions to minimize noise and unnecessary head movements during the scanning process. Resting-state fMRI images were acquired using echo planar imaging (EPI) sequence with the following scanning parameter settings: repetition time (TR)/echo time (TE) = 1000 ms/30 ms, field of view (FOV) = 220 × 220 mm^2^, matrix = 64 × 64, slices = 52, slice thickness = 2.2 mm, slice gap = 0.5 mm, and voxel size = 2 × 2 × 2.2 mm^3^. The scan duration is 360 seconds, and a total of 400 whole-brain images were acquired. Diffusion tensor images were collected using spin-echo planar imaging sequence with the following scanning parameter settings: TR/TE = 3800 ms/72 ms, b-values = 0 and 1000 s/mm^2^, number of diffusion directions = 64, FOV = 220 mm, matrix = 116 × 116, slices = 60, slice thickness = 2.2 mm, and voxel size = 2 × 2 × 2.2 mm^3^. In addition, we acquired routine head MRI sequences (sagittal T1, axial T1, T2, and T2 FLAIR) to exclude intracranial organic lesions.

### Data preprocessing

The dMRI data were preprocessed using FSL pipeline on the FMRIB Software Library (FSL; http://fsl.fmrib.ox.ac.uk/fsl) (16). The main steps are as follows: (1) Conversion of DICOM data to NIFTI format via the MRtrix3 command “dcm2niix”; (2) Denoising and Gibbs-unringing correction; (3) Eddy current and head movement correction based on the “eddy” command; (4) Bias field correction; (5) Non-brain tissues were removed based on the “BET” command, including skull, cerebrospinal fluid, and soft tissues.

The fMRI data were preprocessed using the Graph-theoretical Network Analysis Toolkit (GRETNA) (17) based on the MATLAB platform. The main steps were as follows: (1) Raw DICOM file converted to NIFTI format; (2) Discarding the initial 10 volumes; (3) Slice-timing; (4) Realignment (excluding subjects with a maximum head motion 2.5 mm or rotation > 2.5°). No subjects were excluded in this step; (5) All the images were normalized into the Montreal Neurological Institute (MNI) EPI template and re-sampled to 3 mm³ × 3 mm³ × 3 mm³; (6) All images were smoothed with a 6 mm full-width at half maximum Gaussian kernel (FWHM); (7) Detrending; (8) Regression out nuisance covariates of Friston 24 head motion parameters, white matter, cerebrospinal fluid signals, and global signal; (9) Band-pass filtered (0.01-0.08 Hz); (10) Imaging with framewise displacement (FD) > 0.2 were scrubbed using spline interpolation to minimize the effect of head motion while preserving temporal information in this band. No significant differences in mean FD were found between IGD patients and HC (*p* = 0.346).

### DTI-ALPS index calculation

In this study, we used the DTI-ALPS method developed by Taoka et al. to quantify the glymphatic system-related diffusivity (18). Taoka et al. claimed that the ALPS index is sensitive to head movements during scanning (8). The higher the head movement, the lower the ALPS index. Therefore, we excluded subjects with a maximum head motion > 2.5 mm or rotation > 2.5°. After preprocessing, fractional anisotropy maps, color-coded maps, and diffusivity maps along the x, y, and z axes were generated using the “dtifit” function. Based on previous studies (19), we attempted to place the ROIs on the registered diffusivity maps to reduce subjective influence and ROI depiction time. However, we found that the ROIs covered the CSF areas in most subjects. Therefore, an experienced neuroradiologist used ITK-SNAP software (https://www.itksnap.org) to place four 5 mm diameter ROIs on bilateral projection fibers in the craniocaudal direction (y-axis) and association fibers in the anterior-posterior direction (z-axis) at the level of the lateral ventricle on the FA color-coded map. Based on previous studies (20), we extracted the diffusivity along the x and y axes in the projected fibers and the diffusivity along the x and z axes in the association fibers to calculate the ALPS index, respectively (**Figure 1**). The specific formula is as follows: $ALPS\;index=\frac{mean\left(Dxproj,Dxassoc\right)}{mean\left(Dyproj,Dzassoc\right)}$. Then, we obtained the mean ALPS index by averaging the left and right ALPS values for subsequent analysis.

**Figure 1:**
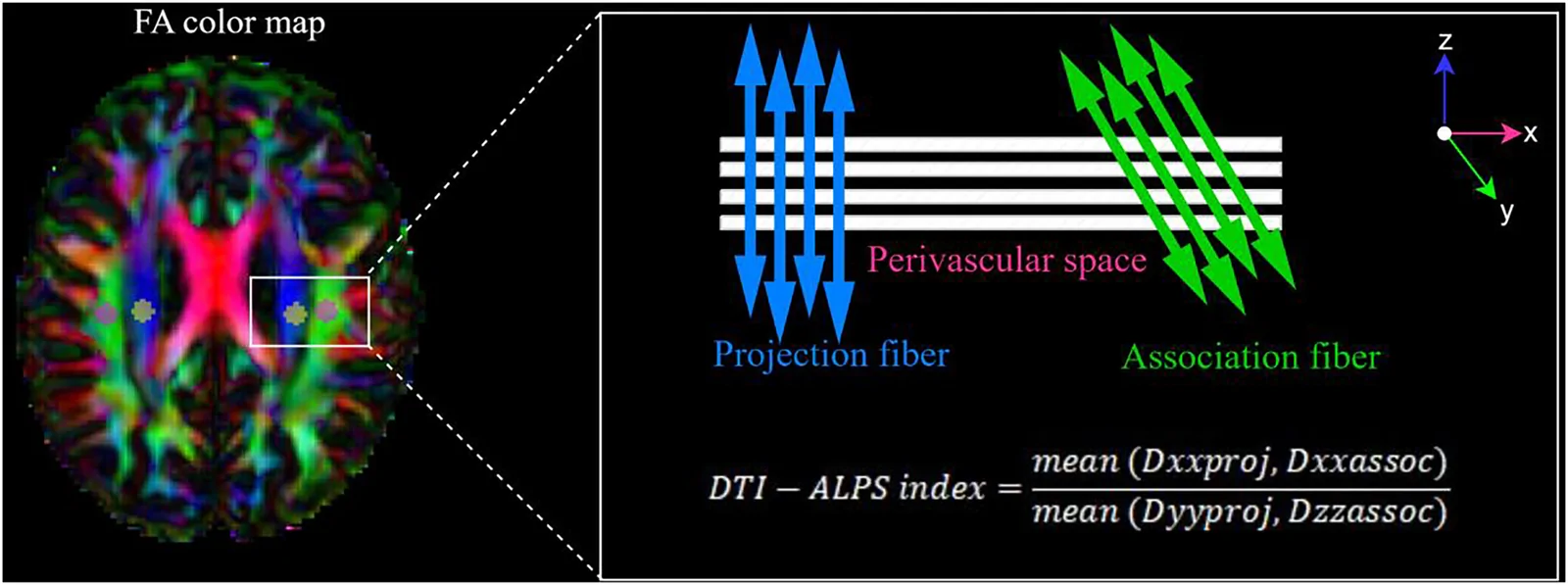
Calculation of index for diffusion tensor image analysis along the perivascular space. FA, anisotropic fraction; *Dxx*, diffusivity along the x-axis; *Dyy*, diffusivity along the y-axis; *Dzz*, diffusivity along the z-axis.

### Brain network construction and graph theory analysis

After fMRI data preprocessing, we performed functional brain network construction and subsequent graph theory analysis for all subjects based on the GRETNA toolbox. First, based on the Brainnetome atlas (21), we divided the whole brain into 246 regions. Subsequently, the mean time course of each region was extracted, and Pearson correlation coefficients for each pair of brain areas were calculated to assess rs-FC. The Fisher’s Z transformation was applied to the obtained correlation values, which could improve the data distribution of the parameter statistics. Different from an arbitrary single threshold, we used a series of specified thresholds (sparsity) to estimate the topological measurements, thus reducing the false positives resulting from low-correlation nodes in the comparisons. The sparsity range was set from 0.05 to 0.40 (step size = 0.01). This range was selected based on three established criteria: (1) ensuring the small-worldness index (σ) > 1.1 for all networks to maintain stable small-world characteristics (22) (23); (2) guaranteeing network sparsity to reduce the influence of spurious correlations (24); and (3) ensuring the network remained fully connected at the lower threshold while preventing excessive randomization and loss of biological plausibility. Considering the interpretation of the results, weighted matrices were binarized at each threshold, retaining only positive connections, and negative correlations were set to zero (22). Finally, a positive binary undirected connected 246 × 246 functional brain network was constructed for each subject.

Next, we explored topological properties at the global level in all subjects, specifically focusing on parameters including global efficiency (Eg), local efficiency (Eloc), and the small-world attribute sigma (σ) in this study. Briefly, Eg and Eloc characterize the efficiency of parallel information transfer in the network and the degree of network fault tolerance, respectively (25). The small-world attribute indicates the degree of small-world organization and represents the optimal connectivity pattern of the network, which is characterized by higher high clustering coefficients (γ) and shorter path lengths (λ) (26). The γ = Cp/Cp_rand_ and λ = Lp/Lp_rand_, where Cp and Lp are the average of the clustering coefficients and average path length coefficients over all nodes, respectively, and Cprand and Lprand are the same averages, but calculated from a matched random network. This random network was created using the Brain Connectivity Toolbox, always preserving the degree of the original network and randomly rewiring each pair of nodes when possible. The definitive γ and λ were the result of averaging 50 repetitions of each parameter with different random networks each time. After that amount of repetitions, the coefficient values remain stable. Finally, the simple quantitative metric of small-worldness (σ) could synthesize the response to these two measurements. In addition, we calculated the area under the curve (AUC) of the topological metrics across this sparsity range to perform the summary measure (27).

### Statistical analysis

All data analyses were performed using IBM SPSS v28 software. Continuous variables are reported as mean ± standard deviation (SD). Normality and homogeneity of variance were assessed using the Shapiro-Wilk and Levene’s tests, respectively. When the homogeneity of variance assumption was violated, Welch’s ANOVA was performed, followed by Games-Howell *post hoc* comparisons.

The demographic and clinical data were analyzed using one-way ANOVA, independent-sample *t*-tests, Kruskal-Wallis test, or Mann-Whitney *U* test for normally or nonnormally distributed continuous data. The chi-square test was used for categorical variables. Significant differences were considered *p* < 0.05.

Analysis of covariance (ANCOVA) was used to analyze the differences for graph theory metrics and the ALPS index (global and bilateral) between IGD patients and HC, with age, sex, education years, HAMA scores, HAMD scores, and mean FD as covariates. Additionally, based on previous studies (19, 28), a PSQI score of 7 or higher was recognized as the presence of a sleep disturbance. Therefore, all IGD patients were further divided into IGD-high PSQI patients (PSQI ≧ 7) and IGD-low PSQI patients (PSQI< 7) for a dichotomized comparison. The Pearson partial correlation analyses were conducted to examine the associations of the ALPS values with graph theory metrics and clinical scores, controlling for age, gender, education years, HAMA scores, HAMD scores, and mean FD. Multiple corrections were performed using the Bonferroni method. In addition, ROC analysis was performed as a secondary exploratory analysis, and the area under the ROC curve (AUC) was calculated to assess the classification performance of the ALPS index for discriminating IGD-high PSQI patients and IGD-low PSQI patients.

Considering the strong correlation between depression, anxiety, and sleep quality, collinearity among PSQI, HAMD, and HAMA scores was assessed in this study using variance inflation factors (VIF) and tolerance, with VIF > 10 and tolerance< 0.1 indicating severe collinearity. In addition, sensitivity analyses were performed after excluding participants with clinically meaningful depressive symptoms (HAMD scores ≧ 20) or anxiety symptoms (HAMA scores ≧ 14).

To examine whether the ALPS index mediates the association between impaired human brain networks and sleep disturbance, we performed mediation analyses. In this process, the brain network measure was included as the independent variable, and the PSQI scores were included as the dependent variable, with the ALPS index acting as the mediator. Mediating effects were calculated based on Model 4 in the SPSS 24.0 PROCESS macro. Then, the bootstrapping approach and bias-corrected 95% confidence intervals (CI) were employed to test the significance of the model mediation effects. Given the cross-sectional design, this mediation analysis was exploratory. The terms “mediator” and “indirect effect” are used in a statistical sense only, and no temporal or causal ordering is implied.

## Result

### Demographic and neuropsychological data

This study included 66 IGD patients and 35 healthy controls. Based on the PSQI scores, 66 IGD patients were classified into 36 IGD-high PSQI patients and 30 IGD-low PSQI patients. The distribution of age, gender, and education years was similar across the groups (*p* = 0.088, 0.585, 0.058). Compared to IGD-low PSQI patients, IGD-high PSQI patients showed increased HAMD, and HAMA scores, which reflected current affective symptom burden. More detailed demographics and measurements are shown in **Table 1**. The overall workflow of this study is shown in **Figure 2**.

**Table 1:** The demographic and clinical data.

Clinical features	IGD-high PSQI (n = 36)	IGD-low PSQI (n = 30)	HC (n = 35)	*F/T/χ^2^*	*P*
Age, mean ± SD (range)	15.67 ± 1.586 (12-19)	14.07 ± 1.874 (12-21)	15.83 ± 5.393 (9-30)	2.493	0.088
Gender, M/F	31/5	25/5	28/7	1.074	0.585
Education, mean ± SD (range)	9.69 ± 2.136 (2-13)	8.03 ± 1.810 (6-15)	9.54 ± 4.334 (4-20)	2.934	0.058
Duration of illness, mean ± SD (range)	3.92 ± 1.811 (1-7)	3.53 ± 1.907 (1-7)	—	0.836	0.406
Gaming time, mean ± SD (range)	9.86 ± 3.331 (3-16)	9.70 ± 3.186 (3-15)	—	0.200	0.842
IAT, mean ± SD (range)	61.22 ± 9.262 (51-83)	61.30 ± 7.693 (50-76)	—	-0.037	0.971
PSQI, mean ± SD (range)	10.44 ± 3.018 (7-17)	3.93 ± 1.680 (1-6)	—	10.526	< 0.001
HAMA, mean ± SD (range)	19.83 ± 9.584 (1-44)	9.47 ± 5.841 (1-20)	—	5.174	< 0.001
HAMD, mean ± SD (range)	28.56 ± 12.989 (3-62)	14.20 ± 6.955 (3-32)	—	5.435	< 0.001
Mean FD, mean ± SD (range)	0.129 ± 0.410 (0.025-0.220)	0.112 ± 0.034 (0.025-0.156)	0.119 ± 0.037 (0.060-0.175)	2.135	0.124

IGD, internet gaming disorder; IGD-high PSQI, IGD with elevated sleep-disturbance symptoms; IGD-low PSQI, IGD without elevated sleep-disturbance symptoms; HC, healthy controls; IAT, the Internet Addiction Test; PSQI, the Pittsburgh Sleep Quality Index; HAMA, the Hamilton Anxiety Scale; HAMD, the Hamilton Depression Scale; FD, framewise displacement.

**Figure 2:**
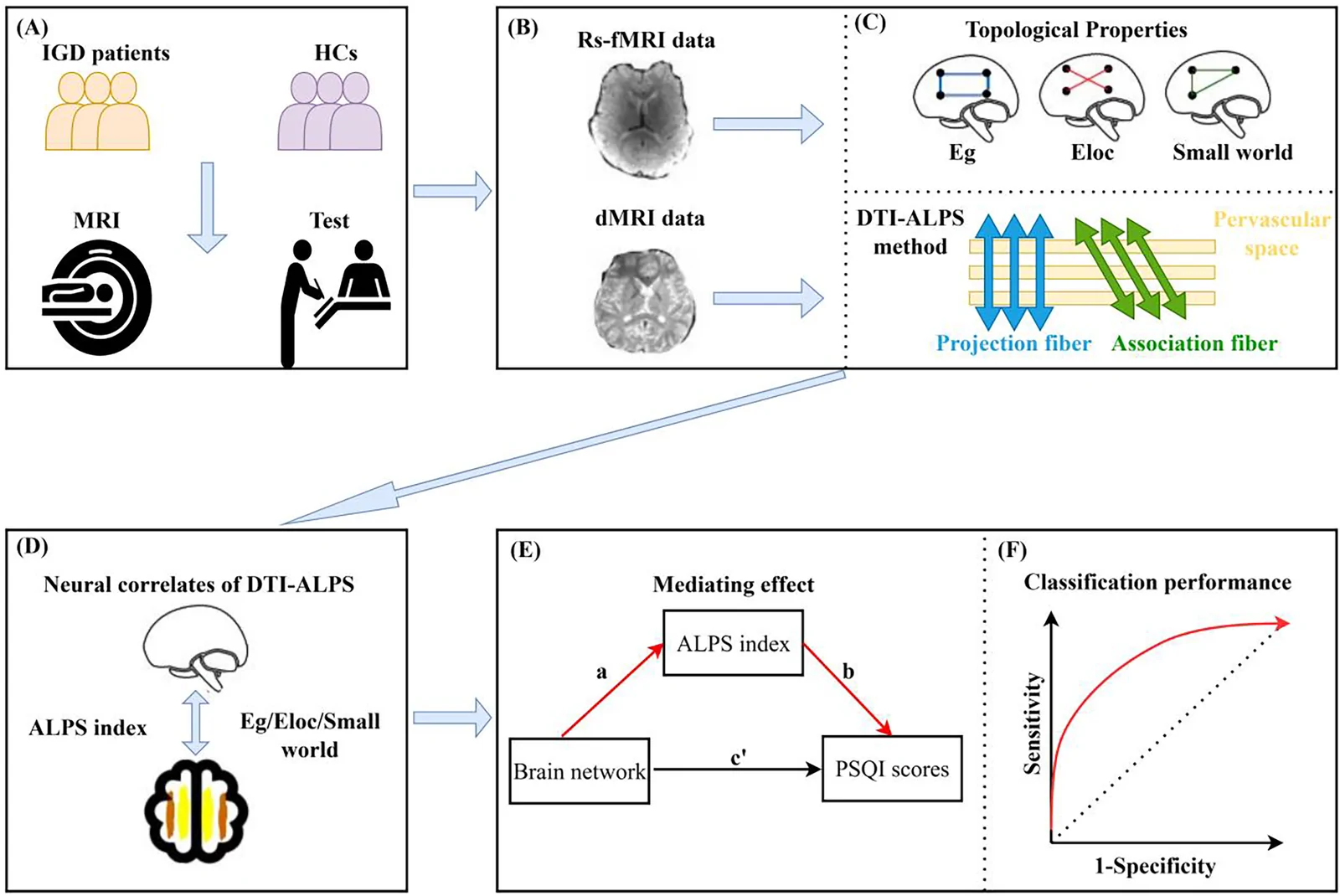
Pipeline of data analyses. **(A)** Collecting magnetic resonance imaging (MRI) and clinical data from all subjects; **(B, C)** The glymphatic system-related diffusivity and network efficiency were calculated based on diffusion images along the perivascular space (DTI-ALPS) index and resting-state functional MRI, respectively; **(D)** The Pearson partial correlation analyses between ALPS index and brain network metrics; **(E)** Mediation analyses of ALPS index on relationship between brain network and PSQI scores; **(F)** ROC analysis was performed, and the area under the ROC curve (AUC) was calculated to assess the classification performance of the ALPS index for discriminating IGD-high PSQI patients and IGD-low PSQI patients.

### Differences in ALPS index among groups

The ALPS index of both hemispheres and the mean ALPS index showed significant differences between the groups. Specifically, compared to the HC, IGD patients showed significant decreased ALPS index in the global brain (*F* = 169.697, *p_bonf_<* 0.001, *partial η^2^ =* 0.641), in the left hemisphere (*F* = 135.416, *p_bonf_<* 0.001, *partial η^2^ =* 0.588), and in the right hemisphere (*F* = 151.451, *p_bonf_<* 0.001, *partial η^2^ =* 0.615) (**Table 2**; **Figure 3**). In addition, the results of the dichotomized comparison further revealed that, compared with IGD-low PSQI patients, IGD-high PSQI patients also exhibited significant decreased ALPS index in global brain and bilateral hemispheres (**Supplementary Table S1**; **Figure 3**).

**Table 2:** The differences of the ALPS index and topological properties between group.

Imaging outcome	IGD patients (n = 66)	HC (n = 35)	*F*	*P_Bonf_*	*Partial η^2^*	Adjusted mean difference (95% CI)
ALPS Index						
Left ALPS	1.309 ± 0.094	1.567 ± 0.120	135.416	< 0.001	0.588	-0.261 (-0.305, -0.216)
Right ALPS	1.301 ± 0.091	1.566 ± 0.123	151.451	< 0.001	0.615	-0.271 (-0.314, -0.227)
Mean ALPS	1.305 ± 0.085	1.565 ± 0.110	169.697	< 0.001	0.641	-0.265 (-0.305, -0.224)
Topological properties						
Eg	0.646 ± 0.008	0.654 ± 0.003	26.366	< 0.001	0.217	-0.008 (-0.011, -0.005)
Eloc	0.769 ± 0.009	0.772 ± 0.007	2.897	0.552	0.030	-0.003 (-0.007, 0.001)
Sigma	1.201 ± 0.087	1.208 ± 0.084	0.184	1.000	0.002	-0.008 (-0.045, 0.029)

IGD, internet gaming disorder; HC, healthy controls; ALPS, the Along the Perivascular Space; Eg, Global Efficiency; Eloc, Local Efficiency.

**Figure 3:**
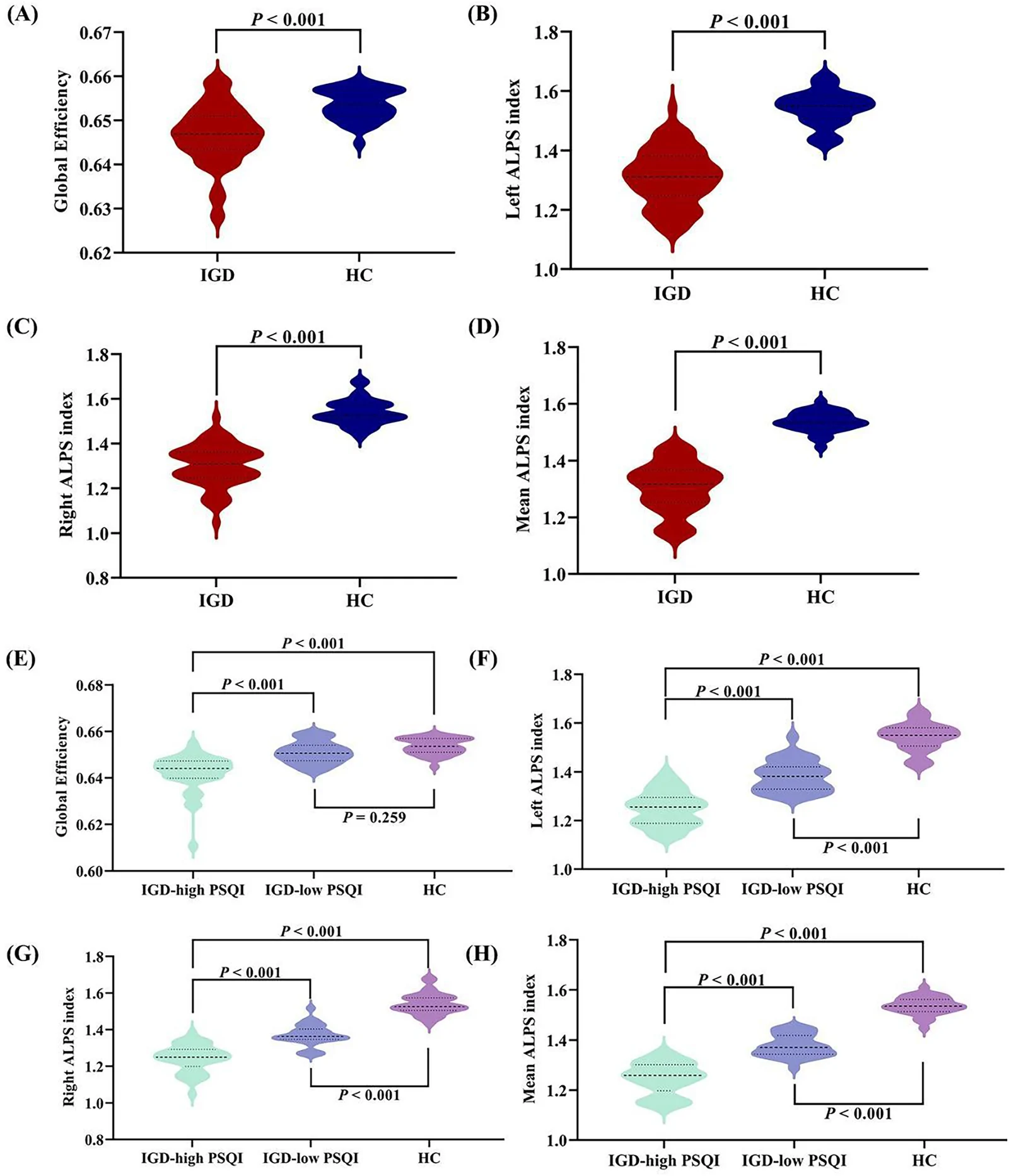
Distribution and group comparison of the ALPS index and graph theory metrics. The panels **(A–D)** comparing IGD and HC groups and panels **(E–H)** comparing IGD-high PSQI, IGD-low PSQI, and HC groups. DTI-ALPS, diffusion images along the perivascular space; IGD, internet gaming disorder; IGD-high PSQI, IGD with elevated sleep-disturbance symptoms; IGD-low PSQI, IGD without elevated sleep-disturbance symptoms; HC, healthy controls.

### Differences in graph theory metrics among groups

The results of the graph theory analysis showed that the functional brain network was impaired in IGD patients. Specifically, compared with HC, IGD patients showed decreased Eg (*F* = 26.366, *p_bonf_<* 0.001, *partial η^2^ =* 0.217). However, there was no statistically significant difference in Sigma (*F* = 0.184, *p_bonf_ =* 1.000, *partial η^2^ =* 0.002) and Eloc (*F* = 2.897, *p_bonf_* = 0.552, *partial η^2^ =* 0.030) between two groups (**Table 2**). In addition, the results of the dichotomized comparison further revealed that, compared with IGD-low PSQI patients, IGD-high PSQI patients also exhibited significant decreased Eg (**Supplementary Table S1**; **Figure 3**).

### Neural and psychological scales correlates of DTI-ALPS index

We further explored how the ALPS index was associated with the human brain network and their relationship in clinical features. The ALPS index was negatively correlated with PSQI score (*r* = -0.542, *p_bonf_* < 0.001), HAMA score (*r* = -0.388, *p_bonf_* = 0.001), and HAMD scores (*r* = -0.334, *p_bonf_* = 0.007) in the IGD patients. No significant correlation was found between the ALPS index and more topological attributes or clinical features in the IGD patients. In addition, we also discovered that Eg was negatively correlated with PSQI scores (*r* = -0.306, *p_bonf_* = 0.015) (**Figure 4**).

**Figure 4:**
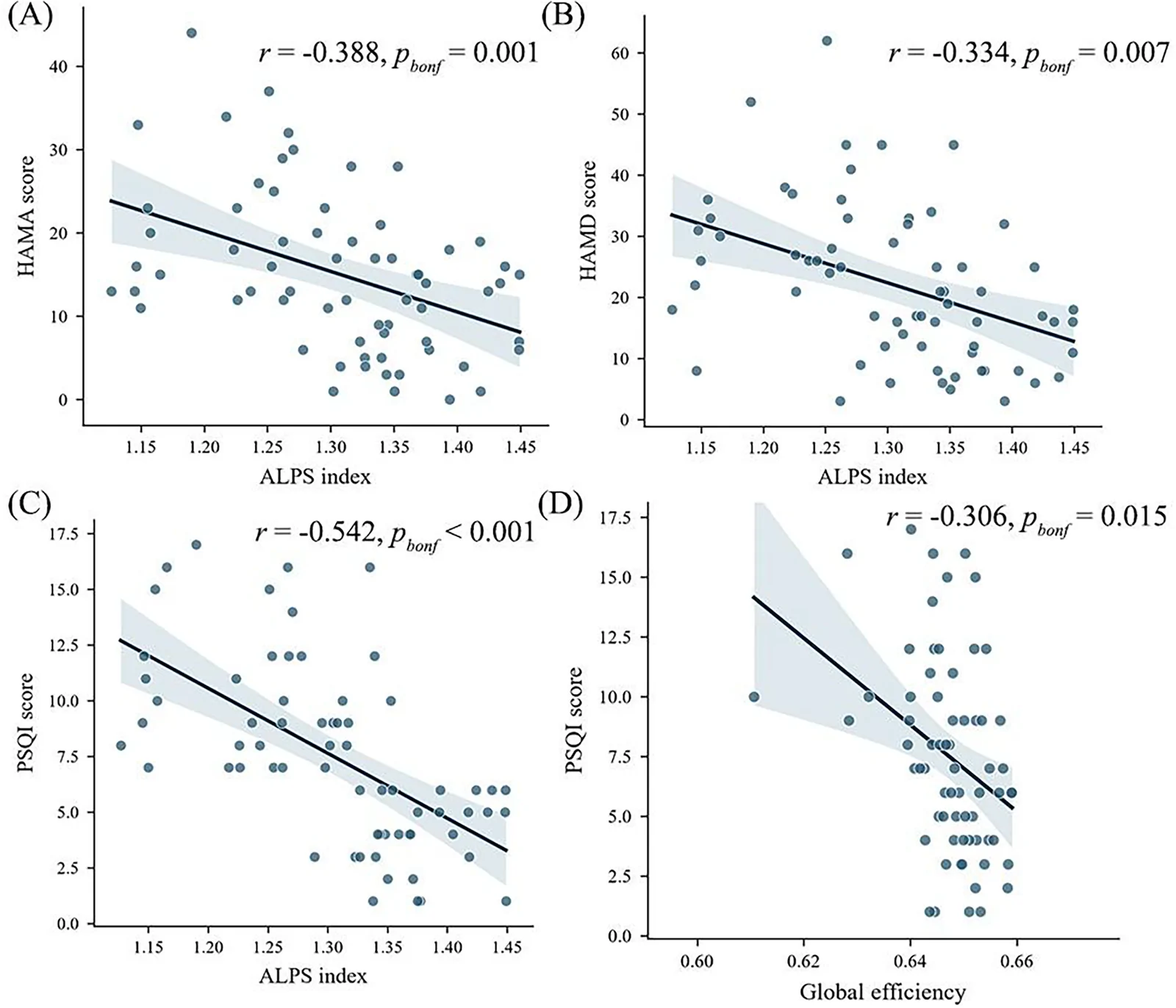
The Pearson partial correlation analysis of ALPS-index and graph theory metrics with clinical features in IGD patients. Plot **(A)** shows HAMA score versus ALPS index (r = -0.388, p = 0.001); plot **(B)** shows HAMD score versus ALPS index (r = -0.334, p = 0.007); plot **(C)** shows PSQI score versus ALPS index (r = -0.542, p < 0.001); plot **(D)** shows PSQI score versus global efficiency (r = -0.306, p = 0.015). DTI-ALPS, diffusion images along the perivascular space; EG, global efficiency; PSQI, the Pittsburgh Sleep Quality Index; HAMA, the Hamilton Anxiety Scale; HAMD, the Hamilton Depression Scale.

### Exploratory mediation analysis and ROC analysis

Given the association among sleep quality, the ALPS index, and human brain network, we performed a mediation analysis. In exploratory mediation analyses, the ALPS index partially mediated the cross-sectional association between Eg and PSQI scores in IGD patients with age, sex, education years, HAMA scores, HAMD scores, and mean FD as covariates (indirect effect = -0.140, mediated effect = 39.4%, 95% CI -0.294 to -0.040) (**Figure 5**). The exploratory ROC analysis showed that the ALPS index could moderately differentiate between IGD-high PSQI patients and IGD-low PSQI patients, with an area under the curve (AUC) of 0.89 (95% CI 0.81 to 0.96) (**Figure 6**). The ALPS index exhibited lower sensitivity (80%) and relatively higher specificity (91%), with a cutoff value of 1.32.

**Figure 5:**
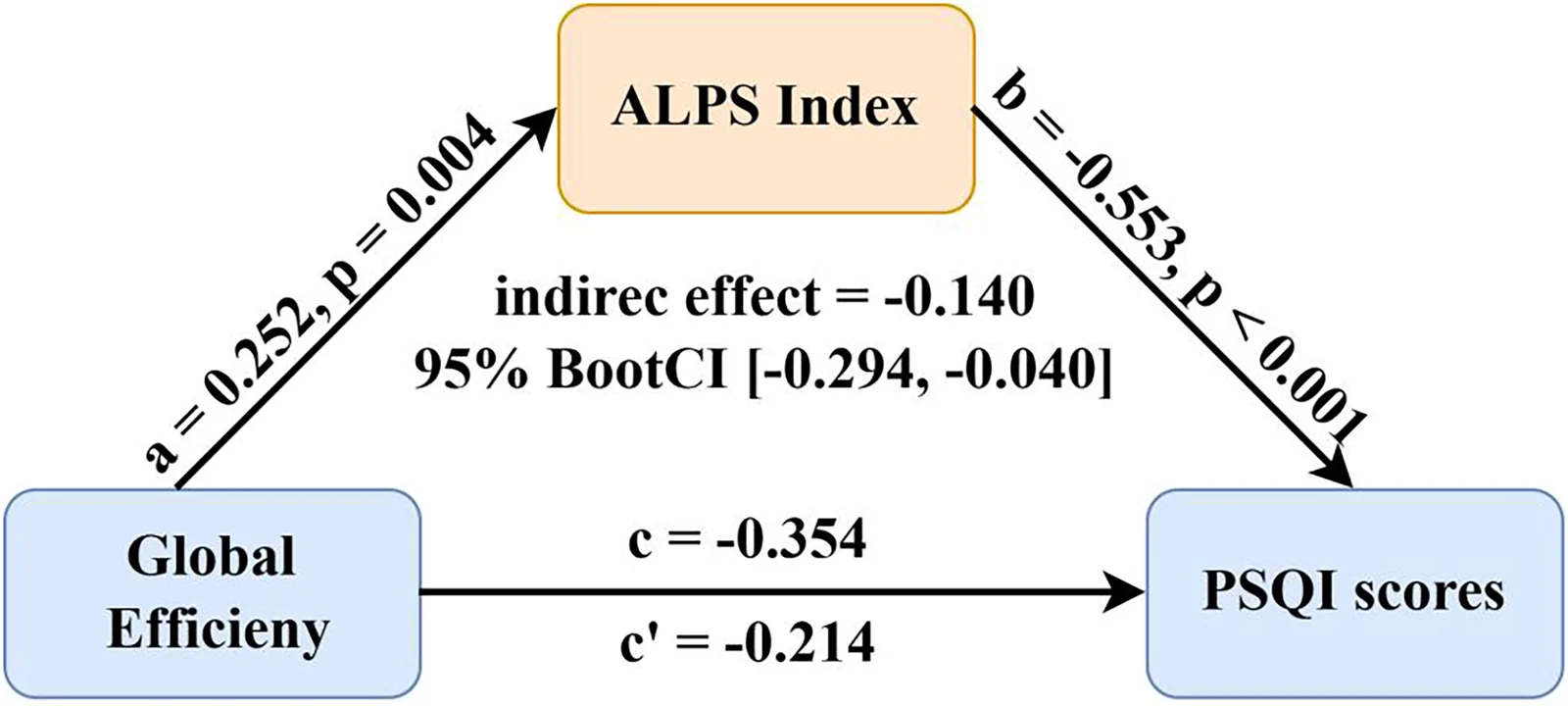
The exploratory mediation analysis among Eg and PSQI scores, with ALPS index as mediator in the IGD patients. DTI-ALPS; diffusion images along the perivascular space; PSQI, Pittsburgh Sleep Quality Index.

**Figure 6:**
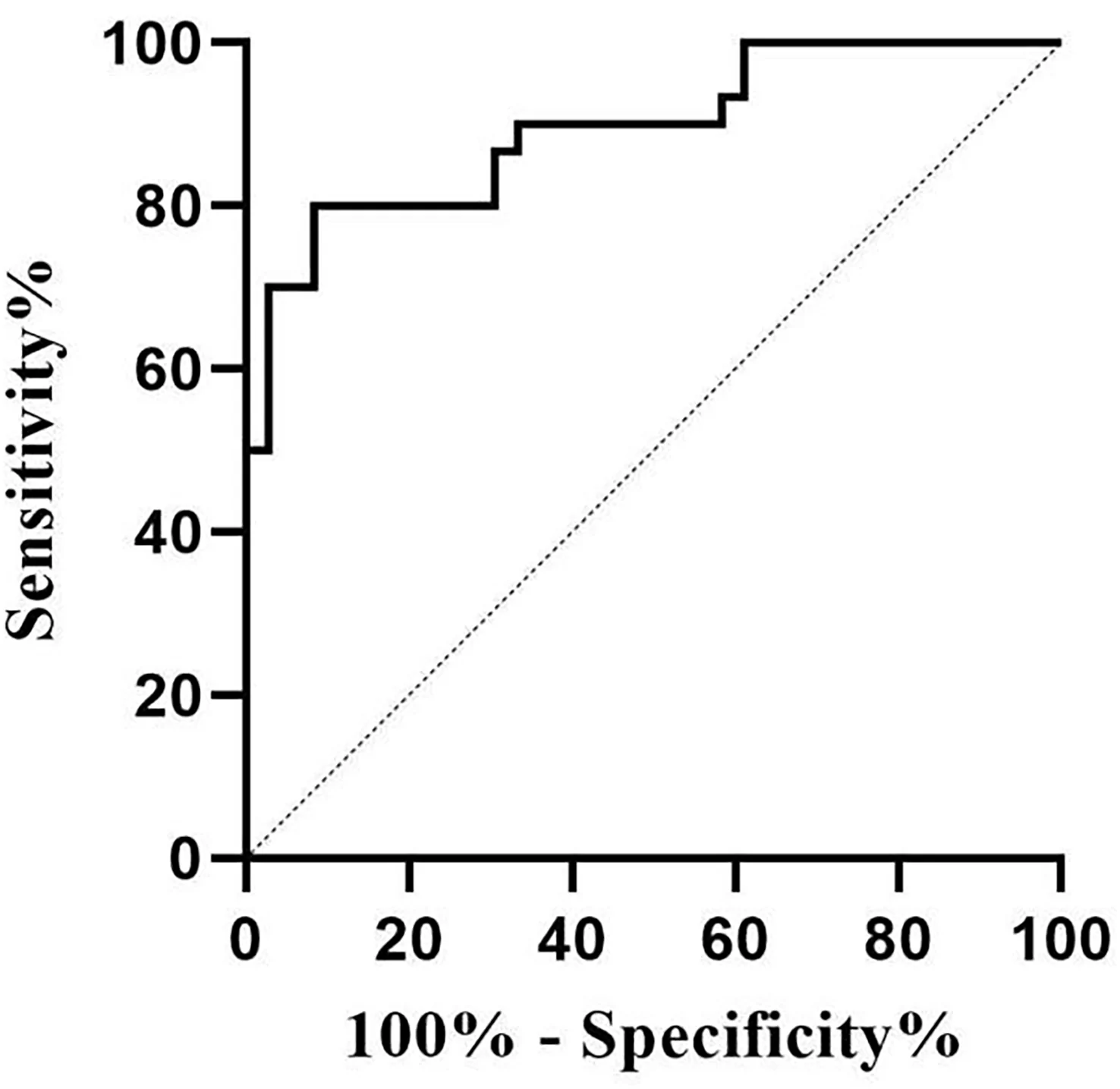
The exploratory ROC curve of ALPS index distinguishing IGD-high PSQI patients and IGD-low PSQI patients.

### Result of collinearity and sensitivity analysis

Considering the strong correlation between depression, anxiety, and sleep quality, this study used PSQI scores as the dependent variable and HAMA and HAMD as independent variables to assess the collinearity among them. The results showed that the VIF and tolerance values were 2.473 and 0.404 respectively, indicating the absence of collinearity. In addition, after excluding participants with HAMD ≧ 20 or HAMA ≧ 14, a total of 25 IGD patients were included in the sensitivity analysis. The results showed that the group difference in the mean ALPS index (*F* = 75.41, *P_bonf_* < 0.001, *partial η^2^* = 0.583) and Eg (*F* = 15.47, *P_bonf_*= 0.001, *partial η^2^* = 0.223) remained significant (**Supplementary Table S2**).

## Discussion

This study investigated the effects of sleep disturbance on IGD patients from the perspective of the glymphatic system-related diffusivity and the human brain network by combining the DTI-ALPS method and fMRI. Specifically, we observed that compared to HC, IGD patients showed significantly decreased ALPS index in the global brain and bilateral hemispheres, which was negatively correlated with PSQI, HAMA, and HAMD scores. In addition, we also observed that IGD patients exhibited significantly decreased Eg. Most importantly, the ALPS index served as a partial mediator in the association between impaired brain network efficiency and poor sleep quality, which may reveal the neuropathological mechanisms of IGD.

IGD patients are frequently plagued by sleep problems, with a large consensus in the literature (29–31). And previous findings also showed that sleep disturbance interfere with glymphatic system-related function. Therefore, this study integrated the DTI-ALPS method to assess the glymphatic system-related diffusivity, which extended the current research in IGD patients. In addition, the ALPS index cut-off in this study was 1.32, leading to an ROC value of 0.89, which could moderately distinguish between IGD-high PSQI patients and IGD-low PSQI patients. However, the groups were defined using a self-reported PSQI and the cutoff and performance estimates were derived from the same sample without internal or external validation. Therefore, these findings do not establish the diagnostic utility or generalizability of the ALPS index. Notably, although the DTI-ALPS method has provided the evidence for glymphatic system-related dysfunction in Alzheimer’s Disease (19), Sleep disturbance (32), and Small Vessel Disease (33) and the effectiveness and reproducibility of the ALPS index was validated by different imaging modalities for evaluating glymphatic systems-related diffusivity (10, 34, 35), numerous researchers claimed that the relationship between ALPS index and glymphatic clearance should be interpreted with caution (36, 37). More recently, Taoka et al. emphasized that the ALPS index should be regarded as a composite biomarker reflecting the interplay among white-matter micro structure, extracellular environment, vascular geometry, and the neurofluid-related tissue environment, rather than as a direct measure of glymphatic function (38). Accordingly, the current findings should be interpreted with caution.

Compared to HC, IGD patients showed decreased ALPS index in the whole brain and bilateral hemispheres, which may suggest impaired glymphatic system-related diffusivity. The glymphatic system facilitates rapid exchange of CSF-ISF within the central nervous system to maintain normal brain function (5). This process relies on AQP-4 in astrocytes to accomplish material exchange (39). Therefore, the loss and polarization disorders of AQP-4 will decrease glymphatic function (40). Previous studies demonstrated that IGD patients were frequently exposed to acute/chronic stress responses (41, 42), which caused HPA-axis dysfunction, further leading to elevated inflammatory factors (e.g., IL-6) (43, 44). This pathophysiologic process has been demonstrated to disrupt AQP4 expression in astrocytes (45, 46), which may lead to glymphatic system-related dysfunction. However, these variables mentioned in the above studies have not yet been measured. Therefore, the hypothesis that the proposed HPA-axis-IL-6-AQP4 pathway may link sleep disturbance in IGD patients to glymphatic system-related dysfunction remains speculative. Further direct biomarker studies are needed in the future to validate our speculation. Notably, compared to IGD-low PSQI patients, this study also found decreased ALPS index in IGD-high PSQI patients. In this context, the negative correlations between ALPS index and PSQI, HAMA and HAMD scores in IGD patients suggested that lower ALPS index may negatively affect sleep quality and emotional fluctuation in IGD patients. However, although IGD patients with formal diagnoses of depression or anxiety were excluded, subclinical affective symptoms were common and strongly correlated with sleep quality. Therefore, the observed group differences may reflect the combined burden of sleep disturbance and affective symptoms, and sleep disturbance cannot be isolated from affective symptom burden in the current sample.

In addition to calculating the ALPS index in IGD patients, we further evaluated human brain networks as a supplementary analysis in this study. Graph theory analysis has provided valuable insights into brain network dysregulation of various neurological disorders (47). This study reported that IGD patients exhibited significantly lower Eg compared to HC. The reduced efficiency of human brain networks indicates impaired capacity for parallel information processing and fault tolerance in IGD patients (25). Therefore, we speculate that this reduced efficiency may be associated with aberrant information communication and resource allocation across brain regions (20). Notably, we identified that Eg was negatively correlated with PSQI scores in IGD patients. Moreover, compared with IGD-low PSQI patients, this study also found decreased Eg in IGD-high PSQI patients. Therefore, this correlation may reinforce the argument that impaired human brain networks are associated with sleep disturbance.

To further explore the intricate relationship among sleep disturbance, the ALPS index, and the human brain network, we conducted mediation analyses. The results revealed that ALPS index partially mediated the association between poorer human brain networks and PSQI scores. Therefore, our findings indicated that global efficiency, PSQI score, and ALPS index showed cross-sectional associations consistent with an indirect pathway. In addition, these discoveries support that simultaneously considering human brain networks could provide a more comprehensive understanding of the neuropathological mechanisms in IGD patients and emphasize the collective impact of the interconnections of human brain networks on the sleep quality. Although the mediation analysis provides substantial support for the proposition that ALPS index serves as a partial mediator in the relationship between impaired brain network and sleep disturbance, it’s crucial to underscore the observational nature of this study. This indirect effect should be considered exploratory and not interpreted as evidence of a causal or directional pathway. In addition, DTI-ALPS is an indirect imaging marker, and the cross-sectional design does not allow causal conclusions. Therefore, future longitudinal and interventional studies are needed to determine whether improving sleep modifies IGD symptoms or ALPS measurements.

## Limitation

Although this study has revealed valuable findings, several limitations are still worth discussing. First, limited by the cross-sectional design, while the current study provides insights into the relationships among sleep disturbance, glymphatic system-related dysfunction, and impaired human brain networks in IGD patients, further longitudinal investigations are required to establish causal linkages. Second, the predominant reliance on the PSQI scale for assessing sleep disturbance in IGD patients introduces potential bias, given its self-report nature. In the future, objective measures such as polysomnography (PSG) should be introduced to provide a more comprehensive characterization of sleep in IGD patients. In addition, we did not include a detailed classification of sleep disturbance, such as difficulty falling asleep and sleep fragmentation. Third, the sample size of this study is small. Thus, the current study is preliminary. Larger samples will be included to further confirm and extend our current observations. Fourth, the DTI-ALPS method is a deductive measure of glymphatic system-related function. The ALPS index does not comprehensively reflect the clearance function of the glymphatic system. Therefore, we need to be cautious about the current findings and their interpretation. Fifth, due to resource constraints, we did not use PET data to measure the levels of toxic proteins in CSF. Therefore, this study lacks direct evidence linking sleep disturbance to glymphatic system-related dysfunction in IGD patients. Sixth, because the ALPS index in this study relied on manual ROI placement, the assessment of the intra- or inter-rater reproducibility was limited.

## Conclusion

The current study revealed abnormalities in DTI-ALPS index and brain network efficiency in IGD patients, which closely correlated with the severity of their clinical features. In addition, our study provided evidence of the association between the ALPS index and the global network efficiency and PSQI scores. Overall, future longitudinal and interventional studies are needed to determine whether improving sleep modifies IGD symptoms or ALPS measurements.

## Data Availability

The original contributions presented in the study are included in the article/**Supplementary Material**. Further inquiries can be directed to the corresponding author.
