# Supplementary material for: Sleep disturbance is associated with reduced DTI-ALPS index and altered functional-network efficiency in internet gaming disorder
Source: Front Psychiatry. 2026 Oct 15;17:1892235. doi: 10.3389/fpsyt.2026.1892235 (PMC13716738; doi:10.3389/fpsyt.2026.1892235)
Supplement: Supplementary file 1 [file SupplementaryFile1.docx]

**Sleep Disturbance Is Associated With Reduced DTI-ALPS Index and Altered Functional-Network Efficiency in Internet Gaming Disorder**

**Supplementary Material**

Figure S1 Participant flow diagram. IGD, internet gaming disorder; IGD-high PSQI, IGD with elevated sleep-disturbance symptoms; IGD-low PSQI, IGD without elevated sleep-disturbance symptoms; HC, healthy controls; PSQI, the Pittsburgh Sleep Quality Index.

Table S1 The differences of the ALPS index and topological properties among IGD-high PSQI patients, IGD-low PSQI patients, and HC.

| Imaging biomarker | IGD patients  (n = 66) | | HC  (n = 35) | *F* | *P_Bonf_* | *Partial η²* | Between-group differences | Adjusted mean diffference (95% CI) | *T* | *P_Bonf_* |
| --- | --- | --- | --- | --- | --- | --- | --- | --- | --- | --- |
|  | IGD-high PSQI  (n = 36) | IGD-low PSQI  (n = 30) |  |  |  |  |  |  |  |  |
| Left ALPS | 1.249 ± 0.070 | 1.381 ± 0.062 | 1.567 ± 0.120 | 114.143 | < 0.001 | 0.708 | IGD-high PSQI  vs HC | -0.327 (-0.380, -0.247) | -15.058 | < 0.001 |
|  |  |  |  |  |  |  | IGD-low PSQI  vs HC | -0.183 (-0.2385, -0.128) | -8.079 | < 0.001 |
|  |  |  |  |  |  |  | IGD-high PSQI  vs IGD-low PSQI | -0.143 (-0.199, -0.087) | -6.235 | < 0.001 |
| Right ALPS | 1.244 ± 0.073 | 1.370 ± 0.056 | 1.566 ± 0.123 | 115.162 | < 0.001 | 0.710 | IGD-high PSQI  vs HC | -0.330 (-0.384, -0.277) | -15.044 | < 0.001 |
|  |  |  |  |  |  |  | IGD-low PSQI  vs HC | -0.201 (-0.256, -0.145) | -8.742 | < 0.001 |
|  |  |  |  |  |  |  | IGD-high PSQI  vs IGD-low PSQI | -0.130 (-0.186, -0.073) | -5.569 | < 0.001 |
| Mean ALPS | 1.246 ± 0.063 | 1.376 ± 0.043 | 1.565 ± 0.110 | 147.284 | < 0.001 | 0.758 | IGD-high PSQI  vs HC | -0.328 (-0.374, -0.281) | -17.069 | < 0.001 |
|  |  |  |  |  |  |  | IGD-low PSQI  vs HC | -0.191 (-0.239, -0.141) | -9.491 | < 0.001 |
|  |  |  |  |  |  |  | IGD-high PSQI  vs IGD-low PSQI | -0.137 (-0.187, -0.088) | -6.742 | < 0.001 |
| EG | 0.642 ± 0.008 | 0.651 ± 0.005 | 0.654 ± 0.003 | 35.915 | < 0.001 | 0.433 | IGD-high PSQI  vs HC | -0.012 (-0.015, -0.008) | -8.154 | < 0.001 |
|  |  |  |  |  |  |  | IGD-low PSQI  vs HC | -0.003 (-0.006, 0.001) | -1.731 | 0.259 |
|  |  |  |  |  |  |  | IGD-high PSQI  vs IGD-low PSQI | -0.009 (-0.013, -0.005) | -5.983 | < 0.001 |
| Eloc | 0.769 ± 0.009 | 0.768 ± 0.011 | 0.772 ± 0.008 | 1.593 | 1.000 | 0.033 | IGD-high PSQI  vs HC | -0.003 (-0.008, 0.003) | -1.211 | 0.686 |
|  |  |  |  |  |  |  | IGD-low PSQI  vs HC | -0.004 (-0.010, 0.002) | -1.723 | 0.265 |
|  |  |  |  |  |  |  | IGD-high PSQI  vs IGD-low PSQI | 0.001 (-0.005, 0.007) | 0.557 | 1.000 |
| Sigma | 1.195 ± 0.076 | 1.207 ± 0.098 | 1.208 ± 0.084 | 0.236 | 1.000 | 0.005 | IGD-high PSQI  vs HC | -0.014 (-0.065, 0.038) | -0.635 | 1.000 |
|  |  |  |  |  |  |  | IGD-low PSQI  vs HC | -0.001 (-0.056, 0.052) | -0.063 | 1.000 |
|  |  |  |  |  |  |  | IGD-high PSQI  vs IGD-low PSQI | -0.012 (-0.067, 0.042) | -0.538 | 1.000 |

Abbreviations: IGD, internet gaming disorder; HC, healthy controls; ALPS, the Along the Perivascular Space; Eg, Global Efficiency; Eloc, Local Efficiency.

Table S2 The differences of the ALPS index and topological properties between groups in the sensitivity analysis

| Imaging outcome | IGD patients  (n = 25) | HC  (n = 35) | *F* | *P_Bonf_* | *Partial η²* | Adjusted mean difference (95% CI) |
| --- | --- | --- | --- | --- | --- | --- |
| *ALPS Index* |  |  |  |  |  |  |
| Left ALPS | 1.350 ± 0.091 | 1.567 ± 0.120 | 54.676 | < 0.001 | 0.503 | -0.220 (-0.230, -0.116) |
| Right ALPS | 1.345 ± 0.069 | 1.566 ± 0.123 | 65.715 | < 0.001 | 0.548 | -0.229 (-0.285, -0.172) |
| Mean ALPS | 1.347 ± 0.0666 | 1.565 ± 0.110 | 75.406 | < 0.001 | 0.583 | -0.224 (-0.276, -0.172) |
| *Topological properties* |  |  |  |  |  |  |
| Eg | 0.648 ± 0.007 | 0.654 ± 0.003 | 15.467 | 0.001 | 0.223 | -0.005 (-0.008, -0.003) |
| Eloc | 0.767 ± 0.010 | 0.772 ± 0.007 | 2.318 | 0.802 | 0.041 | -0.004 (-0.008, 0.001) |
| Sigma | 1.188 ± 0.089 | 1.208 ± 0.084 | 0.942 | 1.000 | 0.017 | -0.023 (-0.071, 0.025) |

Abbreviations: IGD, internet gaming disorder; HC, healthy controls; ALPS, the Along the Perivascular Space; Eg, Global Efficiency; Eloc, Local Efficiency.
